# RETRACTION: Comparative Study on Wound Healing and Infection Between Open and Minimally Invasive Surgical Methods in Pediatric Otolaryngology Surgery

**DOI:** 10.1111/iwj.70620

**Published:** 2025-04-21

## Abstract

**RETRACTION:** WuX. , HuangW. , ZhaoS. , HuangM. , KuangY. , and LiuG. , “Comparative Study on Wound Healing and Infection Between Open and Minimally Invasive Surgical Methods in Pediatric Otolaryngology Surgery,” International Wound Journal 21, no. 3 (2024): e14728, 10.1111/iwj.14728.38385835 PMC10883252

The above article, published online on 22 February 2024, in Wiley Online Library (http://onlinelibrary.wiley.com/), has been retracted by agreement between the journal Editor in Chief, Professor Keith Harding; and John Wiley & Sons Ltd. Following an investigation by the publisher, all parties have concluded that this article was accepted solely on the basis of a compromised peer review process. In addition, further investigation by the publisher found that the authors provided incomplete ethical approval information for the study. The editors have therefore decided to retract the article. The authors did not respond to our notice regarding the retraction.

**RETRACTION:** X. Wu , W. Huang , S. Zhao , M. Huang , Y. Kuang , and G. Liu , “Comparative Study on Wound Healing and Infection Between Open and Minimally Invasive Surgical Methods in Pediatric Otolaryngology Surgery,” International Wound Journal 21, no. 3 (2024): e14728, 10.1111/iwj.14728.38385835 PMC10883252

The above article, published online on 22 February 2024, in Wiley Online Library (http://onlinelibrary.wiley.com/), has been retracted by agreement between the journal Editor in Chief, Professor Keith Harding; and John Wiley & Sons Ltd. Following an investigation by the publisher, all parties have concluded that this article was accepted solely on the basis of a compromised peer review process. In addition, further investigation by the publisher found that the authors provided incomplete ethical approval information for the study. The editors have therefore decided to retract the article. The authors did not respond to our notice regarding the retraction.

